# Pattern of gray matter volumes related to retinal thickness and its association with cognitive function in relapsing–remitting MS

**DOI:** 10.1002/brb3.614

**Published:** 2016-12-20

**Authors:** Jan‐Patrick Stellmann, Hanife Cetin, Kim Lea Young, Sibylle Hodecker, Jana Pöttgen, Diana Bittersohl, Andrea Hassenstein, Timm Oberwahrenbrock, Christoph Heesen, Susanne Siemonsen

**Affiliations:** ^1^ Institut für Neuroimmunologie und Multiple SkleroseUniversitätsklinikum Hamburg‐EppendorfGermany; ^2^ Klinik und Poliklinik für NeurologieUniversitätsklinikum Hamburg‐EppendorfGermany; ^3^ Klinik für AugenheilkundeUniversitätsklinikum Hamburg‐EppendorfGermany; ^4^ NeuroCure Clinical Research Center and Experimental and Clinical Research CenterCharité‐Universitätsmedizin Berlin and Max Delbrück Center for Molecular MedicineBerlinGermany; ^5^ Klinik und Poliklinik für Neuroradiologische Diagnostik und InterventionUniversitätsklinikum Hamburg‐EppendorfGermany

**Keywords:** atrophy, cognition, magnetic resonance imaging, multiple sclerosis, neuropsychology, optical coherence tomography

## Abstract

**Background:**

Neurodegeneration in multiple sclerosis (MS) may be investigated in the visual system as optical coherence tomography (OCT) and magnetic resonance imaging (MRI) allows examining structural integrity in detail. The association between thickness of retinal layers and focal cortical volumes beyond the primary visual system has not been thoroughly investigated.

**Objective:**

To investigate the association between focal cortical volume and thickness of retinal layers.

**Methods:**

Fifty‐four patients (relapsing–remitting MS, mean age 40.5 years, mean disease duration 7.6 years, median EDSS 2) underwent OCT and MRI. The association between focal cortical volume and OCT measurements was investigated with voxel‐based morphometry (VBM). Patterns of association were determined with Yeo's functional network atlas and the Harvard‐Oxford cortical atlas. We used GEE models with cortical volumes from the FreeSurfer parcellation to confirm VBM results. Post hoc, we analyzed the association between OCT, focal cortical volumes, and an extended neuropsychological assessment in a subgroup of 14 patients.

**Results:**

Macular retinal nerve fiber layer (mRNFL) and ganglion cell /inner plexiform layer (GCIPL) showed a robust association with mainly the insular cortex and the cingulate cortex. VBM findings were confirmed with FreeSurfer volumes. The post hoc analysis detected significant correlations between both OCT outcomes and cognition.

**Conclusion:**

Besides the primary visual system, OCT outcomes show a correlation pattern with cortical regions that are known to be important for cognitive performance, predominantly the insula in both hemispheres. Thus, OCT should be further investigated as a marker for neurodegeneration in MS.

## 1 Introduction

Multiple sclerosis (MS) is the most common chronic inflammatory disease of the central nervous system leading to progressive neuroaxonal degeneration and subsequent disability accumulation in young adults (Friese, Schattling, & Fugger, 2014). The visual network has been identified as one of the key functional systems to investigate neurodegeneration in MS. Impairment of the visual system, either by optic neuritis (ON) or by subclinical inflammation and degeneration, is common in MS (Balcer, Miller, Reingold, & Cohen, 2015) and patients rate vision as one of their three most valuable bodily functions (Heesen et al., 2008). As nearly every part of the primary visual system can now be examined in detail, the visual system gained significance in exploring neurodegeneration in MS (Martínez‐Lapiscina et al., 2014). Patient‐reported outcomes like visual quality of life questionnaires and low‐contrast visual acuity charts allow an ecologic valid assessment of visual function, while optical coherence tomography (OCT) and magnetic resonance imaging (MRI) provide detailed information regarding structural integrity and atrophy of the retina and the brain (Martínez‐Lapiscina et al., 2014). Retinal nerve fiber layer (RNFL) thickness has already been used as an OCT endpoint in a multicenter phase 2 trial and will probably be increasingly used due to the easy, fast, and noninvasive assessment (Sühs et al., 2012). Moreover, OCT measures seem to have moderate but consistent correlation with brain parenchymal fraction and brain volume (Balcer et al., 2015; Petzold et al., 2010). The correlation with gray and white matter volume is inconsistent and correlations are generally weak to moderate (Young et al., 2013). In comparison with the macular RNFL (mRNFL) thickness, combined ganglion cell layer and inner plexiform layers (GCIPL) thickness appear to have a better but still only moderate correlation with gray matter volume which is linked to disability in MS (Balcer et al., 2015). Moreover, OCT and MRI are promising tools to detect anterograde neurodegeneration after acute optic neuritis as well as retrograde neurodegeneration due to MS lesions in the posterior visual pathway (Gabilondo et al., 2014; Klistorner et al., 2014). How other functional networks aside from the primary visual system might be associated with neuronal loss within the anterior visual system has not been analyzed in depth (Saidha et al., 2013). Moreover, research is shifting from global atrophy measures such as gray matter volume (GMV) to research on structural and functional connectivity demonstrating the importance of network integrity in MS (Griffa, Baumann, Thiran, & Hagmann, 2013; Kaiser, 2013; Rocca et al., 2016). Investigating focal cortical volume patterns might help to identify regions that are important hubs in functional or structural networks and might contribute to the disability of MS patients. Previous studies indicated, that impairment of visual function might be as well associated with cognitive function in MS (Toledo et al., 2008; Wieder et al., 2013). The purpose of this study was to investigate to what extent OCT measures correlate with cortical areas that are not part of the primary visual system, and to explore if there is a distinctive pattern of association with cortical regions and if these associations might be linked to neuropsychological performance.

## 2 Methods

### 2.1 Patients

Sixty‐eight patients, without (*n *= 30) and with (*n *= 38) MS immunotherapy, aged between 18 and 61 years with a definite diagnosis of relapsing–remitting multiple sclerosis (RRMS) based on the revised McDonald criteria (Polman et al., 2011) and without any other major health disorder were recruited for a prospective observational study at the MS Outpatient Clinic at the Institute for Neuroimmunology and Multiple Sclerosis (INIMS), University Medical Center Hamburg‐Eppendorf, Hamburg, Germany. All participants gave their written informed consent and the local ethics committee approved the study (Ethical Committee of the Board of Physicians in the State of Hamburg, No. PV4405). Baseline data were used for this analysis. All patients underwent cranial MRI and optical coherence tomography (OCT) of the retina including macular and optic disk scan within six months after the MRI. The neurological status as measured by the Expanded Disability Status Scale (EDSS) (Kurtzke, 1983) of all patients was assessed by trained neurologists. Any history of optic neuritis was documented. A subset of *n *= 14 patients underwent an extensive neuropsychological assessment including the following tests: Test battery for attention (TAP—divided attention, alertness, and selective attention), Symbol Digit Modalities Test (SDMT), Paced Auditory Serial Addition Test (PASAT‐3: 3 s version– measuring working memory, sustained attention, and arithmetic capabilities), digit span forward (fw) and backward (bw) for short term and working memory, verbal learning test of the Wechsler Adult Intelligence Scale (WAIS) immediate and delayed recall, Rey‐Osterrieth complex figure test (ROCF) with copy and delayed recall (representing visuospatial abilities and spatial memory), verbal learning and memory test (VLMT), Trail Making Test A (TMT‐A—cognitive processing speed) and B (TMTB—mental flexibility) as well as a semantic and lexical verbal fluency test measured by the Regensburger Word Fluency Test (RWT).

### 2.2 Optical coherence tomography

Spectral domain OCT examination was performed for both eyes of each participant by trained operators using Heidelberg‐Spectralis^®^ SD‐OCT (Heidelberg Engineering, Heidelberg, Germany, Heidelberg Eye Explorer Software version 1.7.1.0). For peripapillary measurements, a 12° circle scan (3.4 mm) around the optic disk was acquired using the built‐in standard protocol (“RNFL‐N”). For assessment of macular volume and individual retinal layers (thickness), a 30° × 25° OCT volume scan protocol (61 B‐scans; ART = 13 frames) centered on the fovea was used.

An independent rater evaluated the quality of the OCT scans according to the OSCAR‐IB Consensus Criteria for Retinal OCT Quality Assessment (Tewarie et al., 2012). Scans with poor quality were excluded from subsequent analysis as well as OCT examinations with incomplete or incorrect scan protocol. For case of patients with one excluded eye‐scan, the other eye‐scan was withdrawn from analysis as well to avoid asymmetry (when comparing both scans). An integrated automated segmentation algorithm (Spectralis Viewing Module version 5.4.7.0; Heidelberg Engineering GmbH) was applied and afterward manually corrected for the presence of boundary detection errors by a single observer for all scans.

The main OCT outcomes of interest were macular RNFL and GCIPL thickness within cylinders of 3 mm centered on the macula. However, to assure that these measurements are meaningful OCT measurements for the purpose of this study, we computed as well other OCT measurements (See supplemental material for details). This was to exclude that other OCT measurements might have a closer association with gray matter volume in our cohort.

### 2.3 MRI

All MRI data were acquired on a 3T MRI scanner (Skyra, Siemens Medical Systems, Erlangen, Germany). The MRI protocol included a magnetization prepared rapid acquisition gradient‐echo (MPRAGE) T1‐weighted sequence (TR/TE = 1900 ms/2.46 ms; TI = 900 ms; 192 slices, slice thickness = 0.9 mm, no gap; FOV = 240 mm; matrix = 256 × 256, flip‐angle 9° for all scans) and T2 sequence (TR/TE = 2800 ms/90 ms; 43 slices, slice thickness = 3.0 mm, no gap, matrix = 256 × 256, FOV = 240 mm).

### 2.4 Image analysis

All images were processed with the FSL‐voxel‐based morphometry (VBM) analysis pipeline (http://fsl.fmrib.ox.ac.uk/fsl/fslwiki/FSLVBM) (Douaud et al., 2007; Good et al., 2001; Smith et al., 2004). Briefly, an automatic brain extraction tool (FSL‐BET) was applied to all T1‐MPRAGE images. Binary brain masks were subsequently inspected and in most cases manually corrected to avoid inclusion of nonbrain tissue. After the application of the corrected masks, gray matter segmentation images were generated using FAST and nonlinearly registered to the MNI 152 standard space (Andersson, Jenkinson, & Smith, 2007). The resulting images were averaged and flipped along the x‐axis to create a left–right symmetric, study‐specific gray matter template. Secondly, all native gray matter images were nonlinearly registered to this study‐specific template and “modulated” to correct for local expansion (or contraction) due to the nonlinear component of the spatial transformation. The modulated gray matter images were then smoothed with an isotropic Gaussian kernel with a sigma of 2 mm. Finally, voxelwise general linear models (GLM) were applied using permutation‐based nonparametric testing, correcting for multiple comparisons across space with the false discovery rate (FDR) method. Threshold‐free cluster enhancement methods were used.

VBM compares differences in gray matter volume across subjects at each voxel. Our analyses investigated if these differences between subjects were correlated with OCT outcomes. Each analysis identified a certain number of voxels within the cortex for which a significant correlation between gray matter volume and the OCT outcome was observed. The distribution of significant voxels in the cortex was specified by 48 cortical regions based on the Harvard‐Oxford atlas of cortical structures (Desikan et al., 2006) as well as by seven main functional systems defined by the Yeo 2011 atlas (Yeo & Krienen, 2011).

In addition, we used FreeSurfer software (Version 5.2.0) for cortical reconstruction and volumetric segmentation (http://surfer.nmr.mgh.harvard.edu/). Again, all brain masks and white /gray matter segmentation were inspected and manually corrected according to the recommendation from the FreeSurfer documentation. We extracted total gray and white matter volumes as well as volumes of cortical areas of the Destrieux parcellation (Fischl, 2004). Corresponding regions of different atlases were defined by major overlap in MNI152 standard space that is, regions might correspond to more than one region in another atlas.

### 2.5 Statistics

Our statistical analysis plan was designed to investigate patterns of association between OCT measurements and cortical volume. An explorative post hoc analysis aimed to explore the association of OCT measurements with cognitive performance in a subset of patients.

We performed descriptive statistics according to the nature of the data as means with standard deviation (SD) or as frequencies and/or percentages. VBM analyses were adjusted for age, gender, and previous ON. The total intracranial volume (TIV) was not added as covariate to VBM as the spatial normalization step already removes differences in TIV (Good et al., 2002). We run separate VBM statistics for right eyes and left eyes. Agreement of VBM from both eyes was estimated by Pearson's correlations. Further, VBM analyses included T2‐lesion volumes as covariate to estimate to what extent previous inflammatory disease activity determines the association between OCT measurements and cortical volume. All VBM analyses were performed for the whole cohort as well as separately for patients without a history of ON (non‐ON). A separated analysis of ON patients was not possible as the small number of patients and the heterogeneous history of previous ON (i.e., right, left, or both eyes affected) would not allow to distinguish between a lost correlation after ON (evidence of absence) or missing statistical power (absence of evidence). We used the fsl implemented Harvard‐Oxford cortical structural atlas to automatically extract the number of significant voxels for 48 cortical areas. For a more global and functionally sounded approach, we used the Yeo atlas to determine significant voxels in seven main functional cortical networks (visual, somatomotor, dorsal attention, ventral attention, limbic, frontoparietal, default mode). All further analyses were performed with Statistics in R 3.2.1.

### 2.6 Patterns of association

Patterns of association were firstly investigated with the Yeo Atlas of seven functional networks. We used bar plots to explore which network shared the highest number of significant voxels in VBM analyses. Differences between networks were post hoc investigated by GEE models correcting for measures from both eyes. Absolute numbers of voxels were used as dependent variable and networks (visual as reference) as independent variables. Next, we explored the patterns of associations in Harvard‐Oxford cortical regions applying the same method. Analyses were performed for all patients as well as only in non‐ON patients. We corrected for multiple testing with the false discovery rate (FDR) method and defined *p*‐values <.05 as statistically relevant. Again, we included T2‐lesion volume as covariate in the models, to determine the strength of association independent from previous inflammatory activity. As a confirmation approach, we investigated if GEE models including cortical volumes from the FreeSurfer cortical parcellation as dependent variables might result in similar association pattern as VBM. The visual agreement between the two methods was estimated within the MNI‐152 standard space.

### 2.7 Post hoc analysis: association with neuropsychological measures

Finally, we investigate in an explorative post hoc analysis the association between OCT measures and cognitive performance for the unselected subset of patients in whom a neuropsychological assessment was available. Again, we used GEE models to investigate the association between OCT measure and neuropsychological tests. Moreover, we explored which cortical regions might be linked to OCT outcomes and neuropsychological tests at the same time by computing standardized estimates β in linear models between cortical regions and neuropsychological tests. Again, both analyses were corrected for multiple testing using FDR.

## 3 Results

### 3.1 Cohort

A total of 54 RRMS patients (Table 1) with a complete brain MRI and retinal OCT data set were included in the analysis. Four untreated and three treated patients were excluded from analysis due to incomplete OCT data. Scans from seven patients showed poor quality in one or both eyes. All MRI data could be analyzed. Four patients had bilateral ON in the past. Single eye ON was documented in 11 patients for the right eye and in four patients for the left eye. The subset of patients with a neuropsychological assessment (*n *= 14) included four patients with a history of ON. In patients without a history of ON (*n *= 32), the correlation between VBM results from both left and right eyes was high (*r *= .85, *p *< .001). If all patients were included, the correlation remained moderate (*r *= .56, *p *< .001).

**Table 1:** Descriptive statistics

	All patients *n *= 54	Non‐ON *n *= 32
Gender		
Male (%)	22 (41)	12 (38)
Female (%)	32 (59)	20 (63)
Age (years)		
Mean (SD)	40.5 (10)	39.9 (9.9)
Disease duration (years)		
Mean (SD)	7.6 (6.7)	7.9 (5.7)
EDSS		
Median (range)	2 (0–4)	2 (0–4)
History of ON		
*n* (%)	18 (36%)	n.a.
Currently treated		
*n* (%)	32 (59%)	24 (75%)
pRNFL (μm)		
Mean (SD)	84.2 (11.2)	86.9 (12.1)
mRNFL (mm³)		
Mean (SD)	0.16 (0.02)	0.17 (0.19)
mGCLIPL (mm³)		
Mean (SD)	0.55 (0.07)	0.56 (0.08)
TMV (mm³)		
Mean (sd)	2.32 (0.12)	2.33 (0.14)
BV (cm³)		
Mean (SD)	1219.8 (114.9)	1209.5 (90.2)
GMV (cm³)		
Mean (SD)	636.9 (56.9)	623.4 (41.3)
WMV (cm³)		
Mean (SD)	582.9 (62.6)	586.1 (55.4)

EDSS, Expanded Disability Status Scale; ON, optic neuritis; pRNFL, peripapillary retinal nerve fiber layer; mRNFL, macular retinal nerve fiber layer; TMV, total macular volume; mGCLIPL, macular ganglion cell/inner plexiform layer; BV, brain volume; GMV, gray matter volume; WMV, white matter volume; SD, standard deviation.

TMV, mRNFL, and mGCLIPL values for 3 mm diameter cylinder centered at the fovea centralis.

### 3.2 Patterns of association in VBM

**Figure 1:**
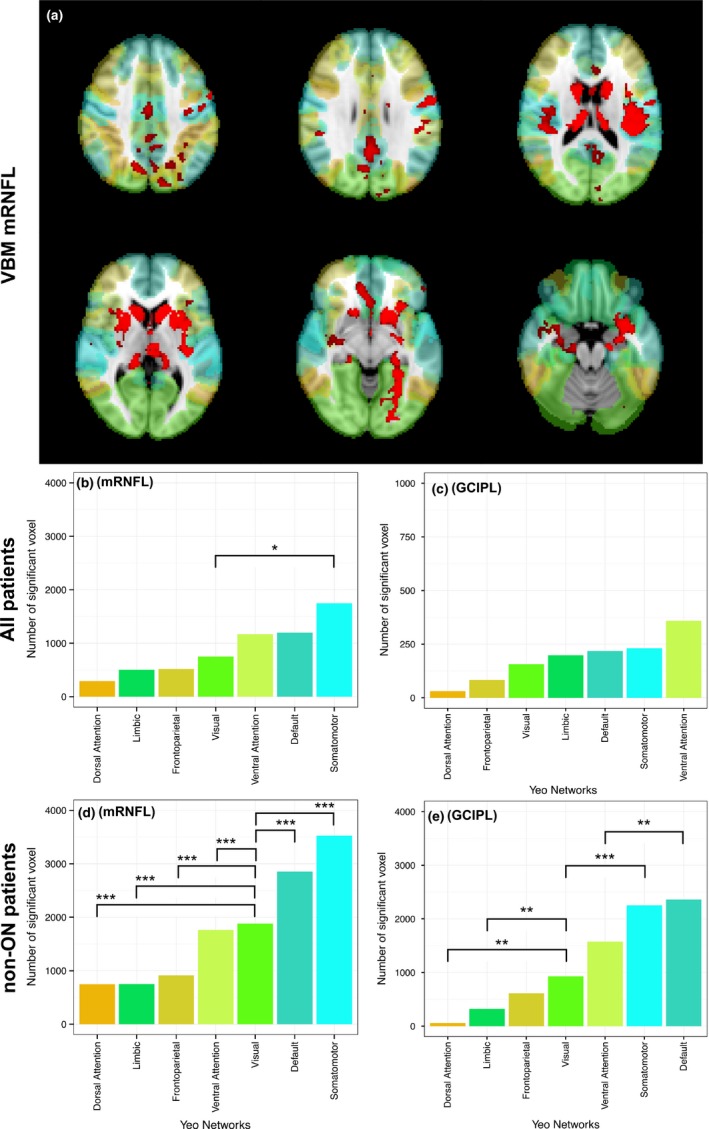
VBM: Significant voxels in functional networks. Association between macular RNFL and GCIPL thickness within a 3 mm cylinder centered at the fovea centralis and functional networks (Yeo cortical atlas). (a) Example of VBM results for mRNFL, colors represent network regions according to the barplots below, significant VBM results are in red, displayed on MNI152 standard. Barplots show median values from left and right eyes. Absolute number of significant voxels associated with macular RNFL (left, b and d) and GCIPL (right, c and e). Results derive from all patients (upper, b and c) and patients without a history of optic neuritis (non‐ON, lower, d and e). Significance of differences (*=*p *< .05, **=*p *< .01, ***=*p *< .001) estimated with GEE models (visual network as reference category)

Based on the Yeo functional network atlas, the highest numbers of significant voxels from VBM analyses in the whole cohort were located in the somatomotor, the default mode, and ventral attention networks (Figure 1). The pattern was similar for mRNFL and GCIPL. Post hoc analyses revealed no significant difference between these three networks in their association with mRNFL (*p *= .07 and *p *= .12, somatomotor network as reference). However, we detected only in the somatomotor network more significantly voxels than in the visual system (*p *= .01). The number of voxels associated with GCIPL did not differ significantly between the seven networks (all *p *> .05). The non‐ON subgroup showed a more pronounced difference between the networks resulting in significant differences of all networks compared to the visual system for mRNFL (all *p *< .001). Investigating GCIPL in the non‐ON group, we found similar results as for mRNFL. More significant voxels were located in the default mode (*p *= .004) and the somatomotor network (*p *< .001) than in the visual network.

**Figure 2:**
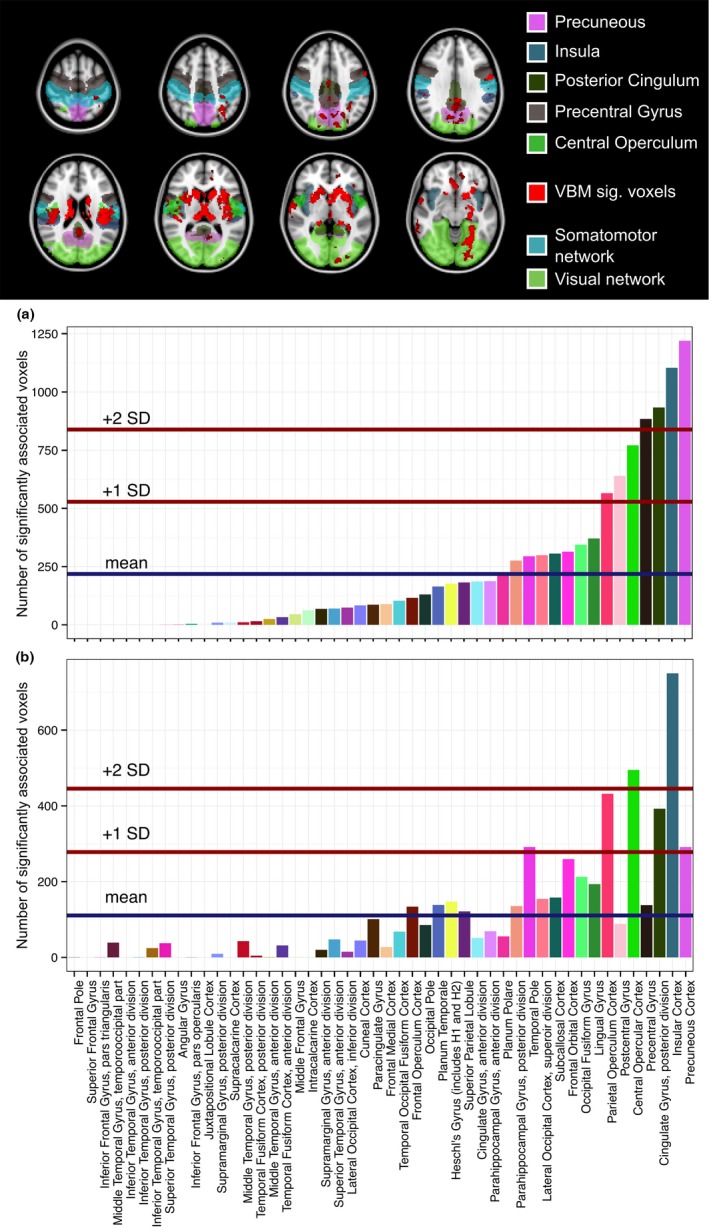
mRNFL VBM: Significant voxels in Harvard‐Oxford regions. Total number of significant voxels in Harvard‐Oxford cortical regions: VBM results of macular retinal nerve fiber layer (mRNFL) from a 3 mm cylinders around the fovea centralis. Top: Overlay of VBM results (red) on MNI152 template. Visual and somatomotor networks as well as the five highest ranking cortical regions are highlighted according to the color codes below. Bottom: Barplots showing absolute number of significant voxels per region (a) non‐ON subgroup, (b) all patients

**Figure 3:**
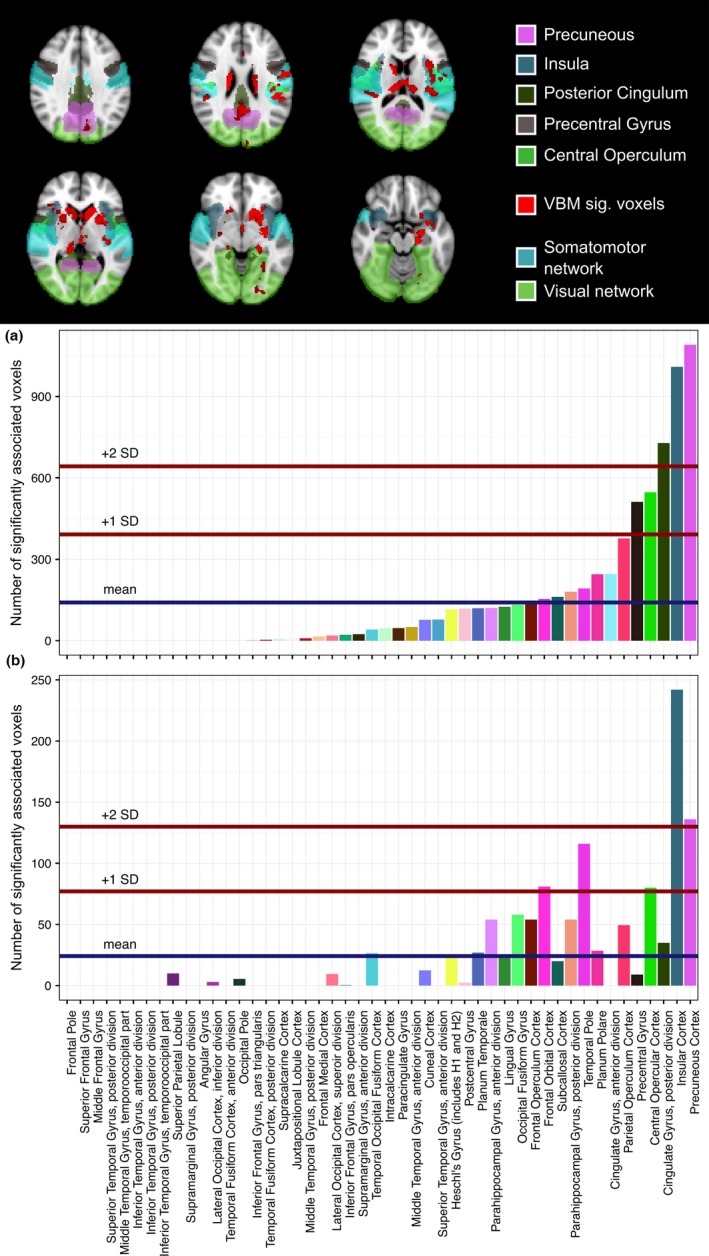
GCIPL VBM: Significant voxels in Harvard‐Oxford regions. Total number of significant voxels in Harvard‐Oxford cortical regions: VBM results of GCIPL from a 3 mm cylinders around the fovea centralis. Top: Overlay of VBM results (red) on MNI152 template. Visual and somatomotor networks as well as the five highest ranking cortical regions are highlighted according to the color codes below. Bottom: Barplots showing absolute number of significant voxels per region (a) non‐ON subgroup, (b) all patients

Based on the Harvard‐Oxford cortical atlas, we observed a close association (two standard deviations above the mean) of the insular cortex with mRNFL (Figure 2) in all patients as well as in the non‐ON subgroup. Moreover, precuneus, posterior cingulate gyrus, and central opercular cortex were associated with mRNFL. However, the association of mRNFL with these cortical regions was weaker in all patients than in those without a previous ON. GCIPL analyses detected a very similar pattern of association with cortical regions (Figure 3). The cortical regions with the strongest association with GCIPL were insula and precuneus.

### 3.3 Confirmation with GEE

Eighteen out of 148 FreeSurfer cortical regions showed a significant positive association with mRNFL while one association was negative (Tables 2 and S1). Eleven of these remained significant if T2‐lesion volume was added as covariate (Figure 5 and Table 3). FDR‐adjusted *p*‐values did not reach significance. The closest association was observed for cortical regions located in the left insula (β = .45), followed by posterior cingulate gyrus (β = .39), superior temporal gyrus (β = .37), and superior occipital sulcus (β = .34). GCIPL thickness was significantly and positively associated with nine cortical regions but significance was lost after FDR correction. Five regions had an inverse association (Table 3). Adjusted for T2‐lesions, six regions with a positive correlation remained significant. Again, the left insula (β = .42) and the posterior cingulum (β = .43) had the closest association. Other regions with high β‐values were the superior occipital cortex (β = .34) as well as the orbitofrontal cortex (β = .32).

**Table 2:** mRNFL and FreeSurfer cortical volumes

Cortical region	Coefficient estimate β	*p*‐value	*p*‐value (FDR)	*p*‐value corrected for T2‐lesion volume	*p*‐value (FDR) corrected for T2‐lesion volume	Corresponding Yeo network	Corresponding Harvard‐Oxford region
lh_G_insular_short	.448	.001	.119	.014	.386	Ventral attention	Insular cortex
rh_G_cingul.Post.ventral	.394	.003	.119	.021	.386	Default	Cingulate gyrus, posterior division
lh_G_temp_sup.Plan_tempo	.365	.004	.119	.017	.386	Ventral attention	Superior temporal gyrus, posterior division
lh_G_Ins_lg_and_S_cent_ins	.361	.016	.256	.084	.664	Ventral attention	Insular cortex
lh_G_occipital_sup	.344	.026	.272	.086	.664	Visual	Occipital pole
rh_G_temp_sup.Lateral	.343	.008	.185	.005	.239	Somatomotor	Superior temporal gyrus, posterior division
lh_S_oc_sup_and_transversal	.318	.012	.225	.002	.160	Visual	Lateral occipital cortex, superior division
lh_S_temporal_inf	.305	.036	.313	.090	.664	Default	Inferior temporal gyrus, posterior division
rh_S_oc.temp_med_and_Lingual	.303	.003	.119	.002	.160	Visual	Lingual gyrus
rh_G_occipital_sup	.302	.020	.256	.053	.524	Visual	Occipital pole
lh_S_oc.temp_med_and_Lingual	.294	.048	.373	.137	.782	Visual	Lingual gyrus
lh_S_circular_insula_ant	.288	.042	.349	.043	.453	Frontoparietal	Insular cortex
lh_G_precuneus	.280	.028	.272	.036	.411	Default	Precuneus cortex
lh_S_front_sup	.270	.024	.272	.118	.758	Dorsal attention	Superior frontal gyrus
rh_S_circular_insula_sup	.266	.004	.119	.024	.386	Ventral attention	Central opercular cortex
rh_S_pericallosal	.228	.009	.185	.022	.386	Frontoparietal	Cingulate gyrus, anterior division
rh_Pole_temporal	.204	.021	.256	.029	.386	Limbic	Temporal pole
lh_S_oc_middle_and_Lunatus	.180	.035	.313	.261	.917	Visual	Lateral occipital cortex, inferior division
lh_Pole_temporal	−.452	.021	.256	.068	.592	Limbic	Temporal pole

Results from GEE models ordered by β, only significant correlations are listed. Corresponding network regions based on main overlap in MNI152 standard space.

**Table 3:** GCIPL and FreeSurfer cortical volumes

Cortical region	Coefficient estimate β	*p*‐value	*p*‐value (FDR)	*p*‐value corrected for T2‐lesion volume	*p*‐value (FDR) corrected for T2‐lesion volume	Corresponding Yeo network	Corresponding Harvard‐Oxford region
rh_G_cingul.Post.ventral	.427	.005	.257	.019	.478	Default	Cingulate gyrus, posterior division
lh_G_Ins_lg_and_S_cent_ins	.421	.014	.319	.026	.478	Ventral attention	Insular cortex
lh_G_insular_short	.372	.023	.319	.128	.726	Ventral attention	Insular cortex
rh_G_Ins_lg_and_S_cent_ins	.370	.017	.319	.049	.524	Ventral attention	Insular cortex
lh_G_occipital_sup	.344	.015	.319	.066	.608	Visual	Occipital pole
rh_S_orbital_med.olfact	.319	.037	.427	.024	.478	Limbic	Frontal orbital cortex
rh_S_oc.temp_med_and_Lingual	.309	.005	.257	.004	.302	Visual	Lingual gyrus
rh_S_circular_insula_sup	.265	.020	.319	.042	.478	Ventral attention	Central opercular cortex
lh_S_interm_prim.Jensen	.265	.031	.388	.132	.726	Default	Angular gyrus
lh_S_front_inf	−.199	.049	.520	.035	.478	Frontoparietal	Inferior frontal gyrus, pars triangularis
rh_G_occipital_middle	−.250	.019	.319	.057	.566	Visual	Lateral occipital cortex, inferior division
rh_G_front_middle	−.313	.024	.319	.024	.478	Frontoparietal	Frontal pole
lh_S_precentral.sup.part	−.391	.020	.319	.003	.302	Dorsal attention	Precentral gyrus
h_Pole_temporal	−.562	.003	.257	.010	.478	Limbic	Temporal pole

Results from GEE models ordered by β, only significant correlations are listed. Corresponding network regions based on main overlap in MNI152 standard space.

**Figure 4:**
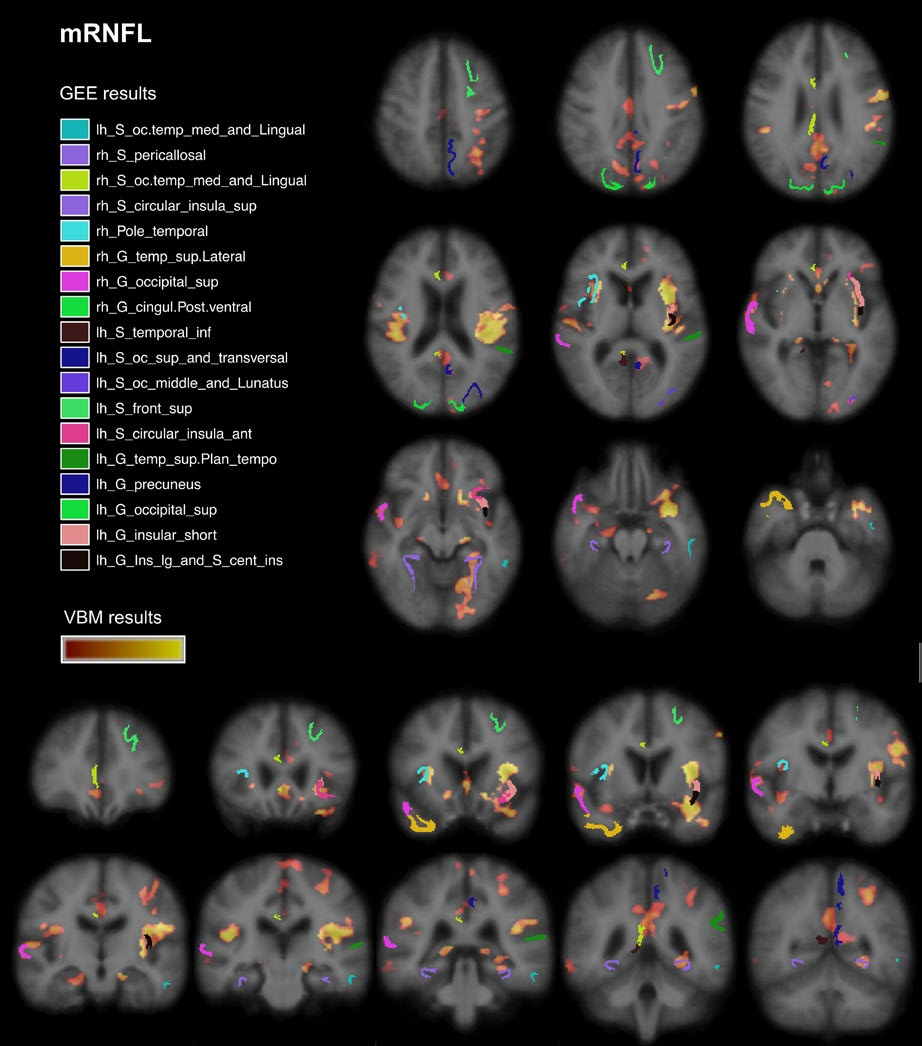
FreeSurfer and VBM regions associated with mRNFL. Results from VBM analysis in pale red (*p *= .04) to yellow (*p *< .001) and significantly associated cortical regions (FreeSurfer parcellation) from GEE models. Displayed on MNI152 standard space

**Figure 5:**
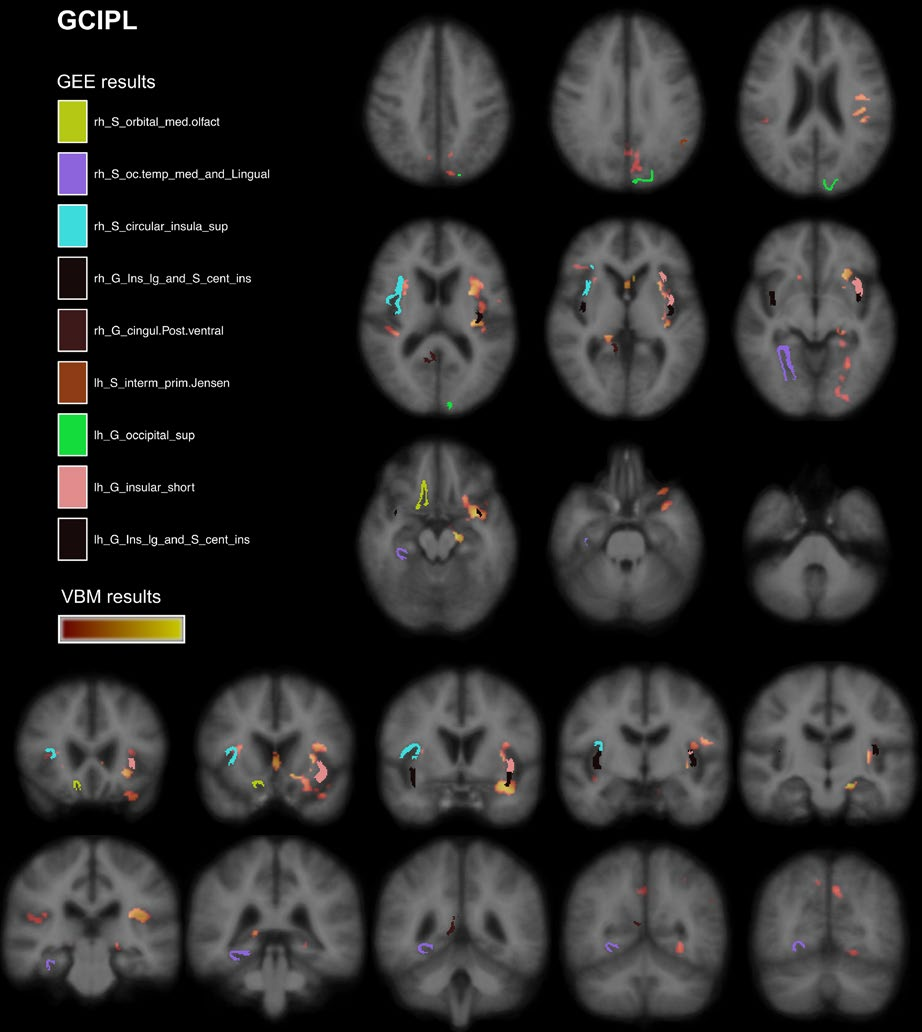
FreeSurfer and VBM regions associated with GCIPL. Results from VBM analysis in pale red (*p *= .04) to yellow (*p *< .001) and significantly associated cortical regions (FreeSurfer parcellation) from GEE models. Displayed on MNI152 standard space

Overall, GEE findings confirmed our VBM results. In the visual system, we observed mainly an association in medial occipital regions including superior parts as precuneus/ cingulate regions as well inferior parts (e.g., lingual gyrus). Outside of the visual system, insula, orbitofrontal, and superior temporal cortex were significantly associated in VBM and GEE analyses. Displaying mRNFL VBM results and significant GEE regions in MNI standard space revealed a direct overlap of significant regions in both insula (Figure 4). In the right hemisphere, we observed an overlap for lateral temporo‐superior cortex , pericallosal sulcus, posterior cingulate gyrus, and orbital medial olfactory sulcus. Figure 5 shows similar findings for GCIPL.

### 3.4 Post hoc analysis: Association with neuropsychological measures

**Figure 6:**
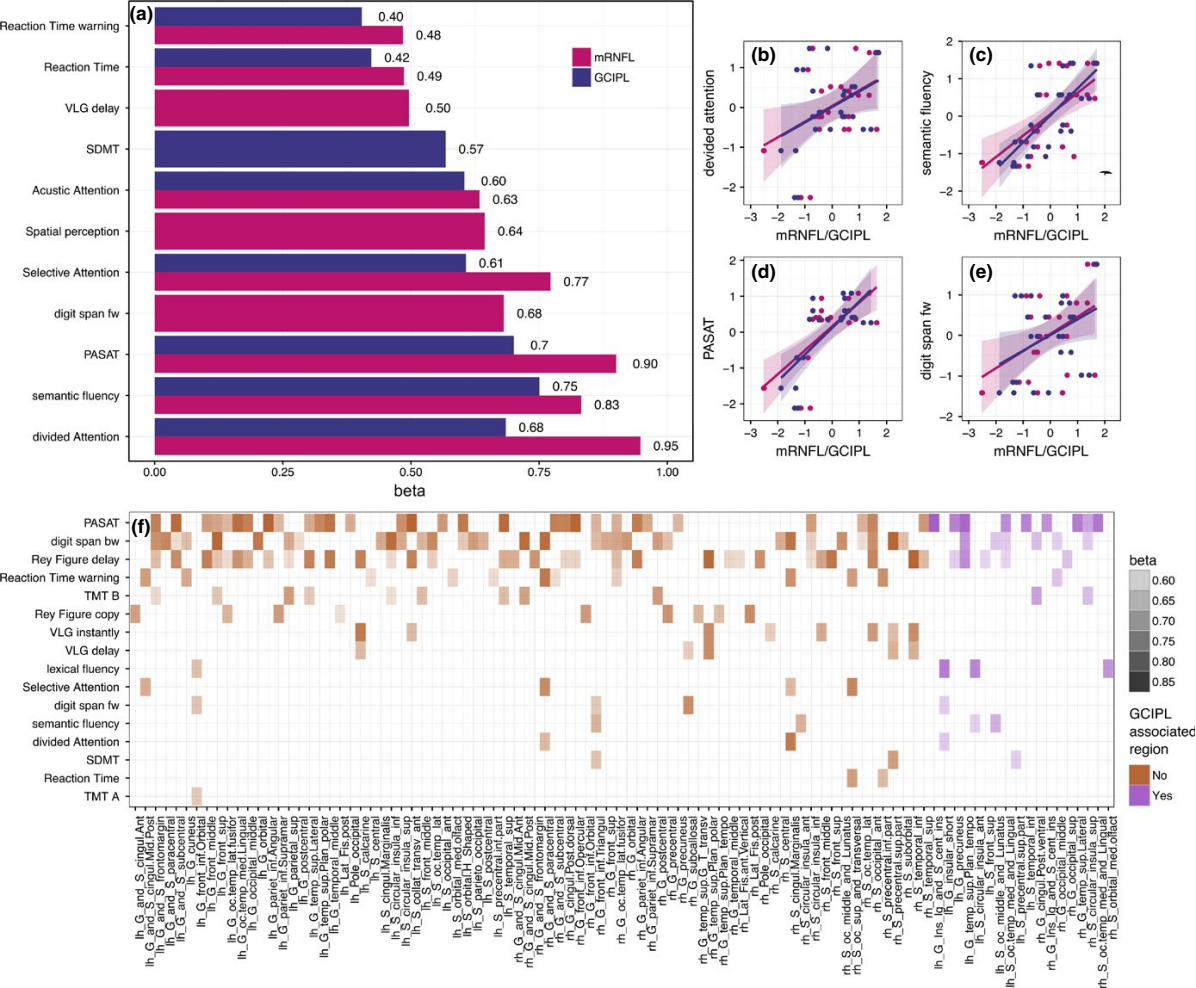
Association between retinal layers, cognitive performance, and cortical regions. (a) Association between cognitive tests (details see method section) and mRNFL/GCIPL thickness. Barplot presents significant standardized regression coefficients β from GEE models of cognitive tests and retinal layer thickness (mRNFL = purple and GCIPL = blue). (b–e) scatterplots with regression lines and its 95%CI (shaded area) for retinal layers and top four associated neuropsychological tests. (f) Cortical regions significantly correlated with neuropsychological tests in GEE models. Heat‐map shows only significant correlations (*p *< .05), shading represents standardized regression coefficient β. Cortical regions that are associated with cognitive performance and GCIPL, are shown in purple on the right side

In the small subset (*n *= 14) of patients with a neuropsychological assessment, we identified a number of neuropsychological measurements correlated with mRNFL and/or with GCIPL (Figure 6): Divided attention, semantic fluency, and PASAT‐3 had the highest β values. mRNFL was associated with 10 tests, while GCIPL was associated with eight. All results remained significant after correcting for multiple testing with FDR (data not shown). Figure 6 also shows the correlation between cognitive tests and cortical regions. Seventeen regions associated with mRNFL or GCIPL were as well correlated with neuropsychological test performance. The closest association with cortical regions was observed for the PASAT‐3 followed by digit span backwards and the delayed Rey figure recall. Temporal superior gyrus, precuneus, and insula were associated with these cognitive tests.

## 4 Discussion

Optical coherence tomography is an established imaging technique to assess neurodegeneration in the primary optic system of MS patients (Balcer et al., 2015). Previous research indicated a moderate but repeatedly confirmed association with global measurements of brain integrity such as white or gray matter volume (Balcer et al., 2015). Here, OCT outcomes show a distinctive correlation pattern with focal cortical volumes pronounced bihemispherically in the insula. Results assessed with VBM were confirmed with a second methodology. We observed a clear link with cortical regions that are already recognized as important integrative regions for brain network function. Moreover, they are well known to be relevant for cognitive function, but to be especially vulnerable to atrophy and connectivity loss in MS (Anticevic et al., 2012; He et al., 2009; Steenwijk et al., 2016). In a small subset of patients, we detected as well an association between OCT outcomes and cognitive performance.

Insula, lateral occipital, posterior cingulate / precuneus, frontopolar and superior temporal cortex were the areas most strongly associated with OCT outcomes in our cross‐sectional study. This pattern shows a good overlap with nonrandom cortical atrophy patterns just recently identified in MS patients compared to healthy controls (Steenwijk et al., 2016). One of these atrophy patterns includes insula, precuneus, and posterior cingulate as cortical regions and was associated with cognitive function. The cortical region with the best correlation with OCT outcomes in our cohort was the insula in both hemispheres. The insula has been identified as being crucial for higher cognitive tasks and attention (Menon & Uddin, 2010). Current concepts interpret the insula as a gatekeeper that assigns priority levels to internal and external stimuli. This appears to manage attention and working memory resources and accordingly influences behavior in healthy controls (Menon & Uddin, 2010). In the ventral attention network, the insula also seems to regulate task assignment to frontal cortical regions accessing executive functions. This appears to be independent from the kind of stimuli (i.e., visual or auditory) or response (Eckert et al., 2009). In MS, insular atrophy is related to T2‐lesion load, functional connectivity, and network efficiency. With increasing T2‐lesion load, the loss of network efficiency is more pronounced in the insula than in other regions (He et al., 2009). Based on several structural and functional MRI studies, inefficient task assignment and recruitment strategies are an established concept to explain cognitive impairment in MS (Rocca et al., 2015). The association between retinal thickness and insula volume in our cohort was considerably robust as findings were reproducible in the whole cohort including patients with previous optic neuritis as well after correcting for T2‐lesion volume and multiple testing. The comparison between the whole cohort and non‐ON patients revealed that the association between OCT outcomes and cortical regions might be blurred by the prevalence of optic nerve impairments such as past optic neuritis. Unfortunately, the sample size was too small to investigate the impact of ON in depth. ON leads to a predominant loss of RNFL thickness in the anterior visual pathway, which translates only to a much lower extend of volume loss in related brain regions (Balcer et al., 2015; Petzold et al., 2010). However, accounting for previous ON in our models was sufficient to preserve the strong association with the insula in the whole cohort. Nevertheless, our findings indicate that OCT might be especially useful as a marker of focal cortical volume loss in patients without a history of optic neuritis and on a group level. The pronounced variability of RNFL thinning after ON restricts its use as an individualized outcome measurement.

Moreover, we observed a close association of OCT outcomes and the posterior part of the cingulate cortex which is as well part of the default mode network (Leech, Braga, & Sharp, 2012). Like the insula, posterior cingulate cortex appears to integrate and connect different brain networks. Dorsal parts are linked to frontoparietal attentional networks and, during attention demanding task, the region seems to be essential for rapid behavioral changes and short reaction times to changing environmental situations (Leech et al., 2012). As attention and information processing speed are the most commonly affected cognitive functions in MS (Langdon et al., 2012; Rocca et al., 2015), prominent atrophy of the posterior cingular cortex might be an important structural correlate. A fMRI study with a go/no‐go response discrimination task detected an increased activation of the precuneus, the posterior part of the cingulum and the insular cortex in MS patients compared to healthy controls (Loitfelder et al., 2014). Previous studies provided already evidence for an association between OCT outcomes, visual function, and neuropsychological measurements (Toledo & Sepulcre, 2008; Wieder et al., 2013). In our small subgroup of patients with a neuropsychological assessment, we performed an explorative post hoc analysis. OCT outcomes appeared to be associated with cognitive tests assessing working memory and processing speed. The two cognitive domains are also typically impaired in MS (Rocca et al., 2015). Our findings indicate that OCT measures might be further investigated as markers of brain volume loss of regions representing important atrophy patterns in MS. Especially, the insula might be used as indicator regions for the overall network integrity in MS (Steenwijk et al., 2016).

Similar to Steenwijk et al. (2016) and due to the cross‐sectional design, our study does not allow to provide an explanation of the pathophysiology leading to the observed correlation patterns. From a pathophysiologic point of view, the association between OCT outcomes and focal cortical volume might be explained by anterograde and retrograde neurodegeneration within the visual system and its connections to major integrating network hubs (Gabilondo et al., 2014). Steenwijk and colleagues called this a “second‐order effect” where focal neurodegeneration leads to accentuate gray matter loss in important network hubs along defined anatomical structures. This hypothesis is supported by studies about the hierarchical network architecture in the brain (Filippi et al., 2013; van den Heuvel & Sporns, 2011). Within this conceptual framework, we assume a progressive loss of neurons in cortical hub regions following neuronal loss in the anterior visual pathway. In how far even a retrograde loss in the retina might be driven by peripheral or hub atrophy remains speculative. The pronounced association of OCT outcomes with cortical regions outside of the primary visual system, for example, the insula, might be explained by the assumption that hub regions receiving input from the primary visual system are especially sensitive to these mechanisms (He et al., 2009).

Our VBM findings could be confirmed with a second analyses method (FreeSurfer). The FSL VBM toolbox uses voxelwise statistics, which might have been compromised by multiple testing even though the family‐wise error rate is controlled and guarantees 95% confidence of no false positive findings (Smith et al., 2004). Moreover, the method is considered reliable and has previously been applied to investigate the association between OCT measurements and selected cortical regions in MS (Gabilondo et al., 2014). However, we aimed to add further evidence using the FreeSurfer software suite to investigate correlations between OCT and the key cortical regions identified by VBM. FreeSurfer's cortical parcellation is based on individual cortical folding patterns to match cortical geometry across subjects instead of using an imported structural atlas (Fischl, 2004). This method immanently has some differences compared to the VBM extracted results, as it avoids multiple testing. However, the individual FreeSurfer parcellation does not match the standardized Harvard‐Oxford structural atlas that we used for VBM analyses.

Here, VBM analyses detected a closer association of mRNFL with cortical volumes than between GCIPL and cortical volumes. However, GEE models of FreeSurfer cortical volumes did not detect a consistent better correlation of focal brain regions with mRNFL than with GCLIPL. The superiority of the mRNFL in VBM analyses is somehow contradictory to previous studies that found a better association of GCIPL with global brain volume measurements (Balcer et al., 2015). Our adjustment of OCT outcomes for individual head sizes might partially explain this discrepancy. Within the VBM processing, all images are spatially normalized to a study‐specific template (Good et al., 2002). This procedure reduces the effect of head size, but does not exclude it. The normalization is not complete as otherwise the perfect fit of individual brains would not allow to compare them. In our cohort, the correlation between head size and mRNFL (*r *= .23, *p *= .02) was higher than for GCIPL (*r *= .17, *p *= .07). Nevertheless, we cannot exclude that VBM results concerning mRNFL might be still biased through remaining head size effects. In contrast, the intracranial volume was easily implemented as covariate in GEE models and we observed a slightly better association of GCIPL with white matter volume than for mRNFL, which is in line with previous studies (Balcer et al., 2015). Moreover, VBM is known to have good test–retest reliability but might be influenced by different scanners, protocols, and segmentation procedures contributing to a reduced comparability between different cohorts (Good et al., 2002). A further distractor might be based on different MRI processing pipelines as, for example, the frequently applied SIENAX algorithm tends to overestimate white matter and underestimate gray matter volume (Lee & Prohovnik, 2008). Another aspect of discrepancy to previous studies might be the retinal segmentation technique, as automatic segmentation algorithms were optimized over the last years. We used an established workflow and all segmentations were performed with the same software version and manually corrected—which does not exclude but minimizes false layer estimates (Brandt et al., 2011; Young et al., 2013; Zimmermann et al., 2013). However, as we were mainly interested in the patterns of association and findings were considerably robust over different methods, outcomes, and adjustment strategies, we believe our results are reliable and still in the range of previously published results.

The generalizability of our study result is limited, as our findings have a risk to be mainly cohort specific. The small sample size and the cross‐sectional design must be seen as the most relevant limitation. Moreover, the missing comparison with healthy controls does not allow to distinguish between MS‐specific or physiological associations. Therefore, it is not possible to draw final conclusions, whether our findings represent a pathophysiological feature of MS or not. However, as our identified patterns of association are in line with previously reported atrophy patterns in MS, we have a substantial indication that the observed findings are relevant to MS (He et al., 2009; Steenwijk et al., 2016). However, further—especially longitudinal—research is necessary to confirm our findings.

We do not procure data from a visual quality of life questionnaire as NEI‐VFQ25, which is a limitation and which would have provided an estimate of the ecologic valid visual impairment of our cohort (Kowalski et al., 2012). Further research should aim to assess the complete visual system in an overarching approach.

## 5 Conclusion

Besides the primary visual system, OCT outcomes show a correlation pattern with cortical regions important for cognitive performance, predominantly the insula in both hemispheres. OCT as an easy, short, and less expensive outcome measurement than MRI should thus be further investigated as a marker for neurodegeneration in MS. OCT might indicate impaired function and structure of important cortical regions as the insula.

## Conflict of Interest

All authors declare no conflict of interest related to the work presented in the paper.
