# Supplementary material for: Pattern of gray matter volumes related to retinal thickness and its association with cognitive function in relapsing–remitting MS
Source: Brain Behav. 2016 Dec 20;7(2):e00614. doi: 10.1002/brb3.614 (PMC5318367; doi:10.1002/brb3.614)
Supplement: Supplementary file 1 [file BRB3-7-e00614-s001.pdf]

Supplemental Material

Comparison of OCT outcomes

Methods

In addition to the main outcomes of interest, mRNFL and GCIPL, we computed the following OCT measurements: total macular volume (TMV), macular RNFL (mRNFL) and mGCLIPL volumes for cylinders with diameters of 6mm, 3mm and 1mm centred on the macula (Supplemental Figure 1A and 1B) as well as mean peripapillary RNFL (pRNFL). We aimed to investigate if alternative outcomes might show a closer association with grey matter volume than the two standard outcomes mGCIPL and mRNFL (thickness, 3 m cylinder).

VBM compares differences of grey matter volume across subjects at each voxel. Our analyses investigated if these differences between subjects were correlated with different OCT outcomes in the same subjects. Each analysis identified a certain number of voxels within the cortex for which a significant correlation between grey matter volume and a selected OCT outcome is observed. We hypothesized, that the number of significant voxels in the cortex, which significantly correlated to OCT measures, represents a measure of the global association with grey matter: The more correlations of voxels the stronger the correlation. An outcome with a close correlation should have a higher number of significant voxels in the cortex than those with a weak association.

In a first step, we compared in how far the absolute number of significant voxels (results for right eyes and left eyes stacked) differ between OCT outcomes by ANOVA and pairwise T-tests. Results were corrected for multiple testing with Bonferroni adjustment. We compared

(a) different layers of the retina: TMV, mGCLIPL, mRNFL and pRNFL

(b) different diameters of measurement cylinders: 1, 3 and 6mm diameters around the fovea

(c) different measures for each outcome: minimal, mean and maximal thickness as well as the volume (mean thickness instead of volume for pRNFL)

Results

Comparing different OCT outcomes, mRNFL and pRNFL did not differ in their association with grey matter (mean significant cortical voxels for both measures 1.4%, p=1.0) but both performed significantly better than TMV (0.9%, p<0.001) and GCIPL (0.2%, p<0.001). Supplemental Figure 1C shows percentage of significant voxels for each Harvard region in all patients. In patients without previous ON, pRNFL was significantly closer associated with grey matter volume than any other outcome (6.0%, p<0.001), followed by mRNFL (2.8%, p<0.001) which was still better than GCIPL (1.8%, p<0.001) and TMV (1.8%, p<0.001, Supplemental Figure 1D). GCIPL and TMV did not differ significantly (p=0.2). Applying the same analyses to different diameters and volume/thickness measures for mRNFL, GCIPL and TMV, we found three and six mm cylinders as similar to pRNFL thickness (p=0.5 – p=1.0) but all more closely associated than 1mm cylinders (all p<0.01, Supplemental Figure 1E) in all patients. pRNFL and mRNFL performed best in non-ON patients. Out of the different macular thickness/volume measures mean thickness and volumes were most closely associated with cortical volume (p<0.001, Supplemental Figure 1G + H). Based on these results, we the mean thickness of 3mm mRNFL and GCIPL as OCT outcomes for all following analyses appear suitable.

Supplemental Figure 1: Different OCT outcomes and VBM

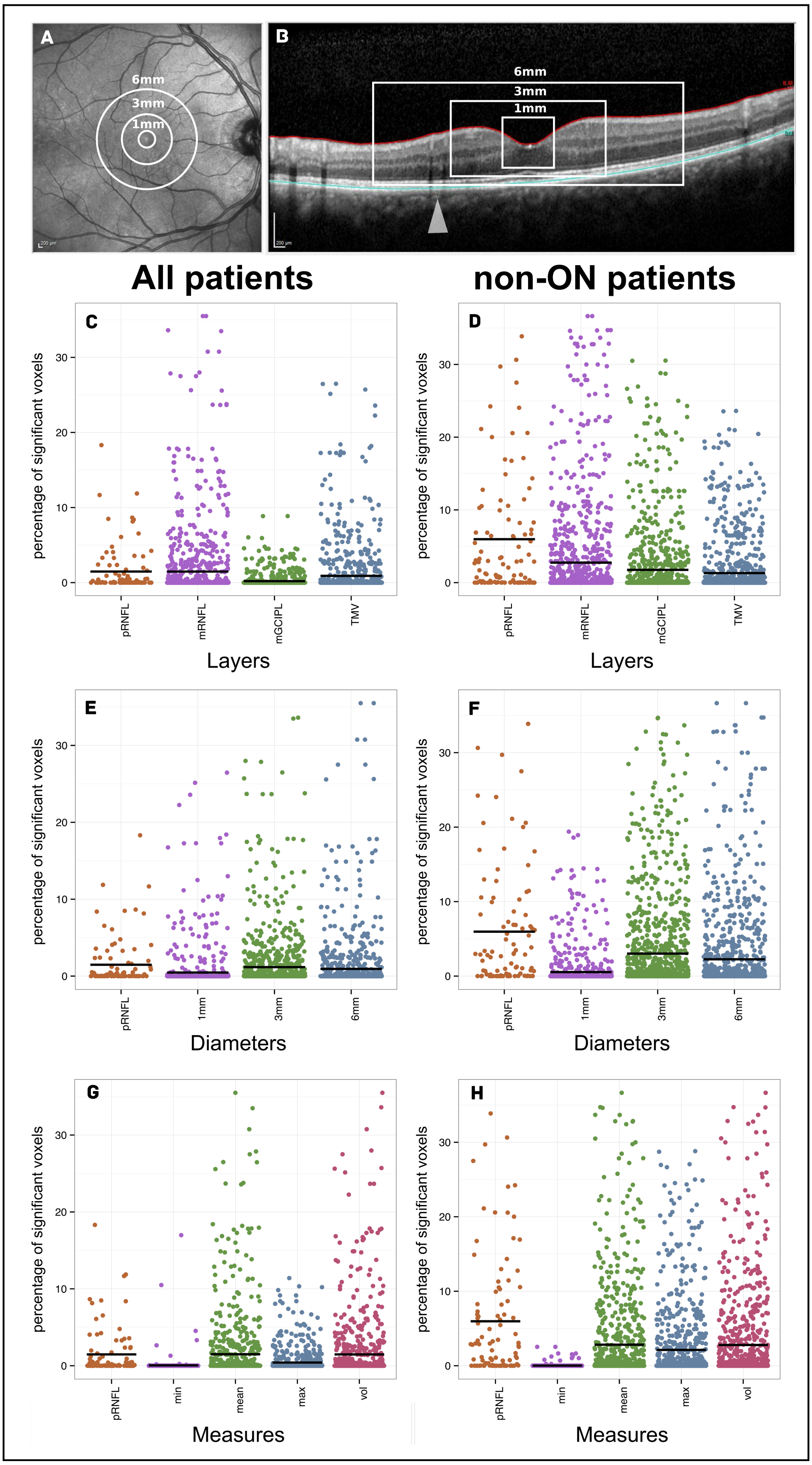

Comparison of different OCT outcomes – **A:** Example macula image with cylinders of 1 mm, 3 mm and 6mm centred at the fovea centralis. Blood vessels are visible outside the 3mm cylinder. **B:** Example of a slice through the fovea centralis of the same patient, again with measurement cylinders. The arrow points towards two vessel artefacts. **C-H** Jitter plots showing percentage of significant voxels in 48 cortical regions in VBM analyses (mean percentage over all regions = black line) Left: whole cohort, Right: Patients without a history of optic neuritis, Layers: macular retinal nerve fibre layer (mRNFL), ganglion cell/inner plexiforme layer (mGCLIPL), total macula volume (TMV) and peripapillary RNFL (pRNFL) from a ring scan around the optic nerve head. Diameters: 1mm, 3mm and 6 mm cylinders of (mRNFL,TMV, GCIPL) and pRNFL. Measures: minimal (min), mean (mean), maximal (max) thickness and volume (vol) of all three macular layers respectively mean thickness of the pRNFL.
